# Financial instruments used by Polish municipalities in response to the first wave of COVID-19

**DOI:** 10.1007/s11115-021-00569-7

**Published:** 2021-11-27

**Authors:** Sławomira Kańduła, Joanna Przybylska

**Affiliations:** Department of Public Finance, Poznań University of Economics and Business, al. Niepodległości 10, 61-875 Poznań, Poland

**Keywords:** COVID-19, municipality, local government, Poland

## Abstract

Studies on the influence of COVID-19 on municipalities are scarce, although it was precisely municipalities that stood in the first line of combat. It is important to estimate the negative budgetary consequences of the crisis for municipalities and to detect potential patterns in the application of their anti-crisis financial instruments. Our study reveals that the initial response of Polish municipalities to the pandemic crisis in this area varied depending on their administrative type, the amount of current income per capita and the population size. However, the correlations between the applied income instruments and expenditures and the above factors were rather weak.

## Introduction

Current research on the COVID-19 crisis focuses in particular on the epidemiological and macro-level socio-economic aspects. The impact of the pandemic on government finances has been widely studied (Cho & Kurpierz, 2020; Hale et al., 2020; Joyce & Suryo Prabowo, 2020). Governments were found to adopt widely varied action strategies (Bouckaert et al., 2020; Raudla, 2021). Few studies, however, analyzed the impact of the pandemic on the economy and finances of local governments (Ahrens & Ferry, 2020; OECD, 2020). At the same time, the problems with the initial responses of municipalities to the COVID-19 crisis are important, because the faster public entities introduce anti-crisis programs, the lower will be the public expenditures that are required to slow down the decrease of economic growth. The identified research gap in the form of the low number of studies that analyzed the reactions of local governments to the COVID-19 crisis inspired us to start research on the subject. The research was conducted based on the examples of Polish municipalities, yet in the era of globalization and pandemic conditions, self-government units in all countries were confronted with the same challenges. The presentation of the Polish experiences contributes to the literature on the responses of local government to various types of crises.

The authors attempted to determine the answers to the following research questions: Q1: What types of budget instruments, both of an income- and expenditure-related nature, were used by municipalities in the first months of the COVID-19 pandemic in Poland? Q2: What was the scale of decrease in current income caused by the application of income-based instruments and what was the expenditure spent on counteracting COVID-19? Q3: What were the sources of financing the expenditures of municipalities on counteracting the effects of COVID-19? In order to obtain a more complex picture of the situation on a local level, certain hypotheses were made (specified in the Research section).

## Background

In Poland, public administration operates on two levels: the state and territorial self-government. The state administrates its territory, establishes legislation, guarantees public security, manages the economy, ensures minimum subsistence for its citizens as well as establishes and finances higher education institutions, theaters and national museums.

Local government is organized into three tiers: municipalities (gmina), districts (powiat) and regions (województwo). As of December 31, 2019, there were: 16 regions, 314 districts and 2477 municipalities. Municipalities and districts have a local nature, regions are the highest level of local government. The municipality is the basic unit of local government. The districts perform tasks exceeding the competencies of municipalities, while regions are responsible for tasks that are beyond the competencies of municipalities and districts. The levels of local government are independent of each other and have different sources of financing.

**Fig. 1:**
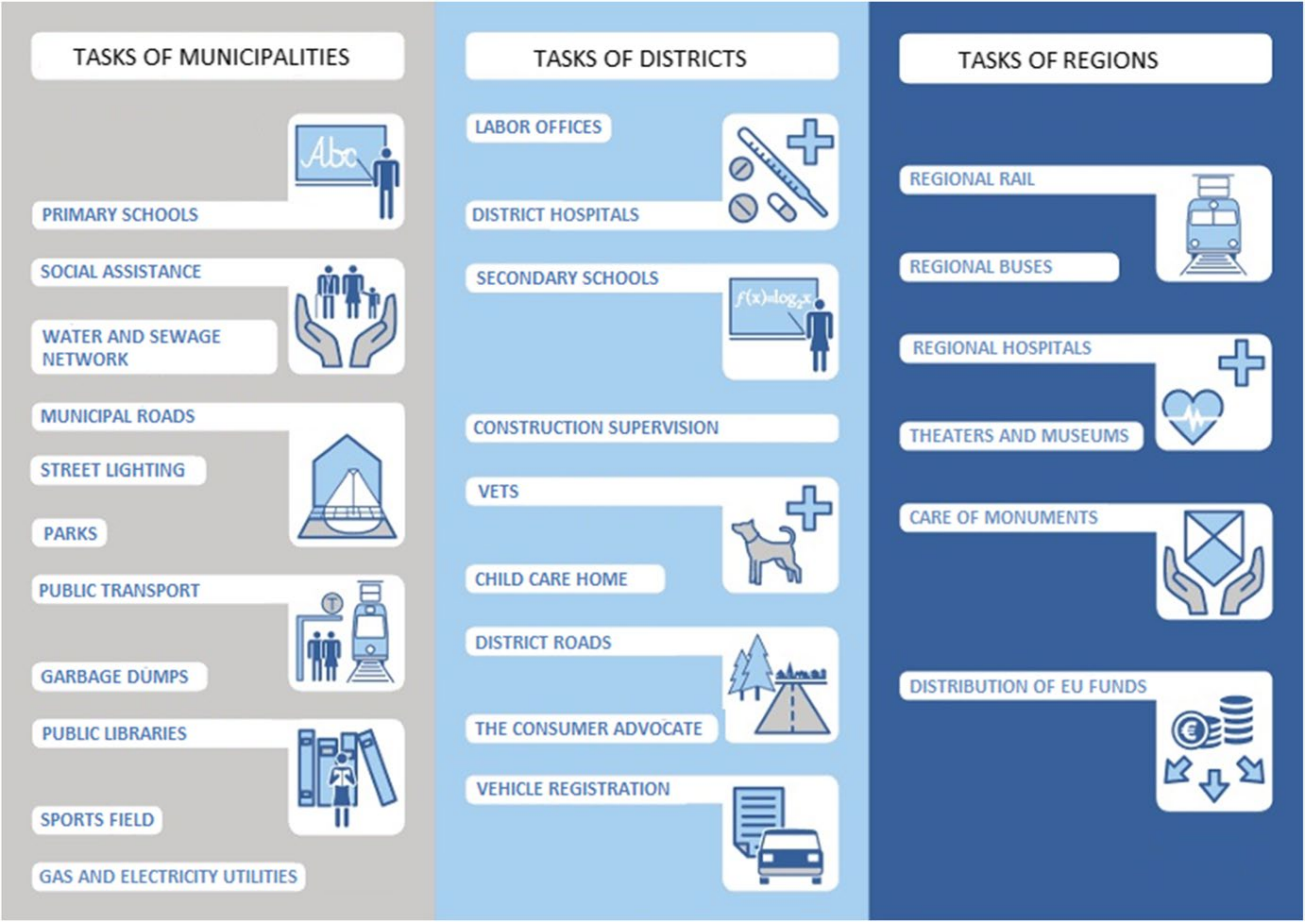
Tasks of local government in Poland. Source: Own elaboration based on (Forum Gminy Wiązowna, 2014)

There are three types of municipalities: urban, which comprise towns; rural, which comprise the countryside and urban-rural, which comprise towns with the surrounding countryside. Some of the urban municipalities have a specific status: one administrative center performs the tasks of both the municipality and the district at the same time. They are called cities with district rights. These units do not form a separate level of local government and are still classified as municipalities. The tasks of local government are presented in Fig. 1.

Among the municipalities, 302 are urban municipalities, including 66 cities with district rights, 638 urban-rural municipalities and 1537 rural municipalities. This study focuses on municipalities, including cities with district rights. All municipalities perform the same compulsory tasks and have the same sources of income but their financial standing is different for objective reasons, such as the number of residents, not for legal status.

In Poland, various entities are responsible for public health. Municipalities promote a healthy lifestyle, help alcoholics and drug addicts, districts establish and supervise district hospitals, and regions are in charge of regional hospitals. The tasks financed from the state budget include clinical hospitals, medical treatment of persons in health emergency situations, sanitary and epidemiological services. Another institution that operates on the state level is the National Health Fund that finances health services (e.g. consultations in clinics, rescue, hospital treatment, all mandatory vaccinations and COVID-19 tests). The necessary means are obtained from health insurance contributions.

**Fig. 2:**
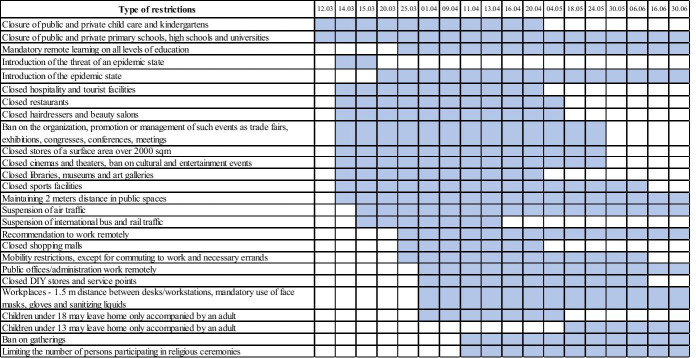
Types of restrictions introduced in Poland to combat COVID-19 before 06.30.2020

Patient zero, i.e. the first person infected with COVID-19 in Poland, was diagnosed on March 4, 2020. By the end of March, 2055 out of 38.4 million inhabitants were infected. At the end of April, this number was 12,640, at the end of May – 23,573, and at the end of June – 34,393. The number of deaths per one million inhabitants was 40 at that time. The types of restrictions that were in force until June 30, 2020, in Poland are presented in Fig. 2.

The first wave of the COVID-19 pandemic in Poland strongly affected the economy. The GDP decreased by 2.8% in real terms, as opposed to the 4.5% increase in 2019. The unemployment rate increased to 6.1%, (at the end of 2019–5.5%). In the first quarter of 2020, there were 35% fewer new jobs, while 120 thousand workplaces were liquidated, which was nearly two times more than in the last quarter of 2019. The pandemic also affected both export and import. In the first 4 months of 2020, the volume of export decreased by 5.3%, and import fell by 5.8%, compared to the same period of 2019.

### Literature overview

The phenomena that have a negative influence on the revenues and expenditures of various entities, including territorial self-government units (TSUs), are referred to in many different ways. As a result, literature mentions economic (or, more narrowly, financial) recession, economic breakdown, slow-down or downturn. The authors of this article decided to use the term “crisis”. Crises differ in several aspects, e.g. in terms of the factors that cause them. They may be short and turbulent or rather “crawling” and difficult to suppress within a short period. They may also differ in terms of their tim of occurrence and duration. Crises may cover limited areas or individual units, but they may also affect many countries or even the whole world.

The financial recession that started in 2007 involved dramatic changes in the financial market that were linked to insufficient liquidity or the insolvency of market players and interventions of public authorities aimed at preventing this situation or mitigating its negative consequences (Bordo et al., 2001; Shahrokhi, 2011). The COVID-19 crisis may be defined as a “rapid, varied depending on the sector, deceleration of business activity in response to a strong, poorly recognized pandemic shock and the resulting, far-reaching administrative decisions” (Wojtyna, 2020). One of its characteristic features is the fact that the uncertainty of the course of the pandemic and its duration causes extremely strong insecurity concerning the course of economic processes. Moreover, it is a specific twin crisis, as health problems have a negative influence on the economy and conducting business activity on the pre-crisis level influences the rate of spread of COVID-19 infections, so that the pandemic and the economy “infect each other” (Wojtyna, 2020).

The COVID-19 crisis is significantly different from the financial recession. However, the reactions of public authorities to the crisis should take into account the experiences in the preparation and implementation of previous anti-crisis programs, as crises always similarly influence the economy, whatever their sources may be. Thus, the recommendations provided by Stiglitz (2020) still remain valid.

The authors wonder what economic consequences the crisis causes for TSU. The recession of 2007 had a negative influence mainly on the budget revenues of self-government units. It is estimated that the previous crisis contributed rather to the decrease in central budget revenues than in the budgets of municipalities because their incomes are less sensitive to the fluctuations of the economic cycle. It was emphasized that the factors that differentiated the influence of the recession on the revenues and expenditures of municipalities were the type of municipality and its size of the population (Špaček & Dvořáková, 2011). One may add to these factors also the model of TSU and the related structure of budget revenues, which may include certain types of income that are sensitive to economic fluctuations. It seems that the same factors may determine the resistance of municipalities to the COVID-19 crisis.

The COVID-19 crisis caused a decrease in production, sales volume and global demand. Research conducted in Poland in mid-2020 revealed that only some of the self-government authorities in economically developed cities (approx. 35%) expected that the pandemic would cause long-term, deep consequences for the local economy. As a result, municipalities did not even attempt to change the priorities and directions of local economic policies (Sztando, 2020). At the same time, the officials argued that the entities affected by the crisis belonged to global capital groups, so the support that they might receive from municipalities would not change their situation. However, small, local producers and service providers were supported nevertheless through small ad hoc tax exemptions. Expectations that self-government will support entrepreneurs and economic growth are quite common (Ahrens & Ferry, 2020).

Another effect of the pandemic was a sudden plunge in budget revenues by several percent (Sztando, 2020). This strongly affected the revenues and expenditures of TSUs (Nemec & Špaček, 2020), although this influence varied (Gordon et al., 2020). The factors that cause the differences in the influence of the COVID-19 crisis on TSUs in individual countries include (Allain-Dupré et al., 2020): the degree of decentralization, the income structure (Chernick et al., 2021) and its ability to absorb fiscal stress, the fiscal stability of TSUs as well as the forms and effectiveness of the aid received from state authorities. Moreover, the negative consequences of the COVID-19 crisis are more severe for small municipalities, large cities, municipalities that have public transport, and, finally, municipalities that are attractive for tourists (Nemec & Špaček, 2020). In the USA, communities with tourist attractions also suffered strong negative consequences (Gordon et al., 2020). The first studies conducted in the Czech Republic and Slovakia (Nemec & Špaček, 2020) suggest that the COVID-19 crisis, as opposed to the recession of 2007, will be more painful for local budgets than for central budgets. Not only are tax revenues falling, but also parking fees, public transport fares, off-license permit fees and the revenues from the lease of community real estate. This trend is accompanied by additional expenses on ensuring sanitary safety, supporting entrepreneurs, etc. (Gordon et al., 2020). The actual influence of the COVID-19 pandemic on the revenues and expenditures of municipalities is still difficult to predict, as the experiences from previous crises show that prolonged recession ultimately leads to cuts in municipality budgets. Such situations usually occur with a 1–2 year delay (Blöchliger et al., 2010). In such conditions, without the support of the central government, TSUs will not be able to support economic growth in a significant way nor satisfy the needs of residents (Ahrens & Ferry, 2020).

In response to the COVID-19 crisis, municipalities may use several strategies (Maher et al., 2020): reduce discretionary expenditures; reduce the investment costs and expenses on ongoing maintenance; limit the time of providing services or reduce employment. They may also actively counteract the consequences of the crisis in two ways. The first one consists of using income policy instruments: reducing local taxes and levies, fees for services and the use of property (Dvorak, 2021; Sztando, 2020). The second method consists of using the expenditure policy instruments of the TSUs and providing business entities with support in the form of so-called incentive packages (Ito & Pongeluppe, 2020).

The TSUs is an element of the public finance sector, so phenomena related to crises penetrate the self-government finance system. During the previous recession, some authors (Poniatowicz, 2014) reported that the revenues of TSUs were decreasing systematically. This led to an increase both in the budget deficit and in the amount of their debt (Reinhart & Rogoff, 2011). In Poland, this trend coincided with large-scale self-government investments co-financed from the public aid funds of the European Union in the 2007–2013 financial perspective. (Guziejewska, 2010).

Municipalities counteract crisis phenomena and stimulate economic growth with the use of instruments of local anti-crisis policy. These instruments may be classified according to various criteria. The authors of this study used the classification presented in Table 1.

**Table 1:** Instruments of anti-crisis policy of municipalities

Revenue policy instruments	Expenditure policy instruments
Fiscal policy: e.g. tax exemptions, lowering tax rates, extending payment periods, write-off of tax arrears	Direct support for entrepreneurs: e.g. loan suretyship
Instruments related to fees for services: e.g. exemption from the fee for a permit to sell alcohol	Expenses on improving safety
Pricing policy: e.g. lowering the fees (prices) for using community premises, reducing the fees for municipal services (e.g. water supply, waste disposal, kindergartens)	Expenses for informational and educational purposes
	Investment expenditures

Source: Own study

### Research

#### Methodology and characteristics of the community

The article was written based on a literature review and survey conducted by the authors among Polish municipalities (as of June 30, 2020) concerning the actions aimed at supporting business entities and residents that had been introduced by municipalities in the first four months (March–June) of the COVID-19 pandemic. The aim of the paper is to answer the research questions specified in the Introduction. The following hypotheses were made:

- H1: The type of revenue instruments used depends on the administrative type of the municipality, its population, the amount of current budget revenues per capita and the amount of expenses related to actions aimed at counteracting COVID-19.
- H2: The type of expenditure instruments used depends on the administrative type of the municipality,
- H3: The administrative type of the municipality influences the sources of financing the increased budget expenses related to counteracting the effects of COVID-19.

Data was collected on an electronic survey form that was filled out by the respondents independently. The survey was sent to all municipality offices in Poland and covered a total of 2477. Ultimately, 1874 (*N*) municipalities responded to the survey (the return rate was 75.7%). It is a representative sample of the entire population of municipalities in Poland. The characteristics of the respondents are presented in Table 2.

**Table 2:** Characteristics of the respondents

Specification	*N*	*%*
Administrative type		
Rural municipality	1129	60.2
Urban and rural municipality	469	25.0
Urban municipality	223	11.9
City with district rights	53	2.8
Number of residents (2019, thousands)		
Below 10	1190	63.5
10–20	410	21.9
20–30	124	6.6
30–40	47	2.5
40–50	30	1.6
50–100	42	2.2
Above 100	31	1.7
Current revenue per capita (2019, thousand USD)		
below 0.75	444	23.7
0.75–0.88	116	6.2
0.88–1.00	118	6.3
1.00–1.13	318	17.0
1.13–1.26	456	24.3
Above 1.26	422	22.5

Exchange rate: 1 USD = 3.9806 PLN.

Source: Authors’ study

Most of the municipalities declared a decrease in revenues due to granted exemptions, allowances, discounts and other activities aimed at counteracting the effects of COVID-19 on a level below 12.6 thousand USD. The expenses incurred on activities connected with combating the consequences of the pandemic were also usually below 12.6 thousand USD (Table 3).

**Table 3:** Decrease in revenues and the expenditures amounts in municipality budgets (thousand USD)

Decrease in revenues			Decrease in expenditures		
	*N*	*%*		*N*	*%*
Up to 12.6	1306	69.7	Up to 12.6	935	49.9
12.6–25.1	207	11.0	12.6–25.1	368	19.6
25.1–50.2	134	7.2	25.1–50.2	203	10.8
50.2–75.4	64	3.4	50.2–75.4	107	5.7
75.4–100.5	41	2.2	75.4–100.5	105	5.6
100.5–125.6	19	1.0	100.5–125.6	76	4.1
125.6	103	5.5	125.6–251.2	54	2.9
			Over 251.2	35	1.9

Source: Authors’ study

### Results

The responses of municipality authorities to the COVID-19 crisis were varied. Municipalities used various types of financial instruments. Research demonstrated that the majority of municipalities did not establish subjective exemptions from local taxes and charges. Only 26.0% of municipalities used this instrument. Less than half of municipalities extended the periods for the payment of local taxes and charges (41.0%). Approximately half of all units granted allowances on local taxes and charges (50.4%) (Table 4).

**Table 4:** Types of financial instruments used by the municipalities

Specification	No		Yes	
	*N*	*%*	*N*	*%*
Subjective exemptions from local taxes and charges	1386	74.0	488	26.0
Local tax and fee allowances	929	49.6	945	50.4
Extensions of the period of payment of local taxes and charges	1106	59.0	768	41.0

Source: Authors’ study

In the first months of the COVID-19 pandemic in Poland, the payments of local charges or the related arrears were divided into installments in a small number of municipalities. Such preferences usually applied to real property tax (16.8%) and tax on means of transport (6.4%). Few municipalities divided the payment of local charges such as the location charge (0.7%), climate fee (0.6%), advertising fee (0.5%) and the dog owners’ fee (0.6%) or the related arrears into installments (Table 5).

**Table 5:** Division of local charges or related arrears into installments

Specification	No		Yes	
	*N*	*%*	*N*	*%*
Real property tax	1559	83.2	315	16.8
Tax on means of transport	1755	93.6	119	6.4
Agricultural tax	1793	95.7	81	4.3
Forest tax	1841	98.2	33	1.8
Marketplace fee	1856	99.0	18	1.0
Local charge	1860	99.3	14	0.7
Climate fee	1863	99.4	11	0.6
Advertising fee	1864	99.5	10	0.5
Dog owners’ fee	1862	99.4	12	0.6

Source: Authors’ study

As far as cities with district rights are concerned, very few units limited the administrative enforcement of financial liabilities. Those who decided to do so more often decided not to enforce the amounts due from natural persons (16.1%) than from legal entities and organizational units without legal personality (15.2%). Tenants of community premises were also rarely released from the obligation to pay rent. Such exemptions were made in 25% of municipalities for commercial premises and 9% of municipalities for residential premises (Table 6).

**Table 6:** Limitation of administrative enforcement and exemptions from the obligation to pay rent

Specification		No		Yes	
		*N*	*%*	*N*	*%*
Limitation of administrative enforcement of financial liabilities (cities with district rights only)	Natural persons	1572	83.9	302	16.1
	Legal entities and organizational units without legal personality	1590	84.8	284	15.2
Exemptions from the obligation to pay rent for tenants of community premises	Residential premises	1826	97.4	48	2.6
	Commercial premises	1406	75.0	468	25.0
	Natural persons	1706	91.0	168	9.0
	Legal entities and organizational units without legal personality	1742	93.0	132	7.0

Source: Authors’ study

Few municipalities lowered the fees for the use of municipal infrastructure, e.g. for water supply, waste disposal or subsidized utility bills. Municipal receivables under civil law that were deferred or divided into installments included mainly rent (12.3%), less often lease (9.6%) and the fee for the use of municipal land (8.5%). Residents were also seldom released from the obligation to pay for public nurseries (12.2%) and kindergartens (11.4%) (Table 7).

**Table 7:** Types of non-tax revenue instruments used by the municipalities

Specification		No		Yes	
		N	%	N	%
Fees for the use of municipal infrastructure / utility bills subsidized	Water supply fee	1852	98.8	22	1.2
	Waste management fee	1857	99.1	17	0.9
	Subsidies on bills	1860	99.3	14	0.7
Civil law liabilities due to the municipality deferred or divided into installments	Rents	1643	87.7	231	12.3
	Leases	1694	90.4	180	9.6
	Fee for the use of municipal land	1714	91.5	160	8.5
Exemptions from the obligation to pay fixed fees for kindergartens and public nurseries for residents	Kindergartens	1660	88.6	214	11.4
	Public nurseries	1646	87.8	228	12.2

Source: Authors’ study

In search for patterns in the responses of municipalities to the COVID-19 crisis, the authors analyzed whether, and to what extent, the administrative type of the municipality was linked to the type of the revenue instruments used (H1). To verify it, an analysis with *χ*^*2*^ Pearson tests was conducted. It revealed that municipalities differed in terms of using exemptions, allowances, and deferrals of payments of local taxes and charges *p* < .001. Exemptions or allowances were most often used in urban municipalities and least often in rural ones. On the other hand, the municipalities that most often used payment deferrals were cities with district rights as well as urban municipalities, while rural units used these instruments least frequently. However, these differences were not very significant (Cramer’s V coefficient was lower than 0.03). The surveyed municipalities also differed in terms of the policies for the division into installments of: real property tax *p <* .001, tax on means of transport *p* < .01 and agricultural tax *p <* .05. The first two of these taxes were divided into installments in urban municipalities and cities with district rights, while the agricultural and forest taxes – in rural municipalities, which is not surprising, considering the specificity of the tax base of these levies. No differences were found between municipalities in terms of the division of other charges or the related arrears into installments (Table 8).

**Table 8:** Correlations between the type of municipality and the type of revenue instruments used

Specification		Rural municipality	Urban and rural municipality	Urban municipality	Cities with district rights	*χ*^*2*^	*df*	*p*	*V*
Subjective exemptions from tax and local fees		218 (19.3%)	161 (34.3%)	90 (40.4%)	19 (35.8%)	69.67	3	.000*	.19
Local tax and fee allowances		475 (42.1%)	280 (59.7%)	146 (65.5%)	44 (83%)	90.37	3	.000*	.22
Extensions of the period of payment of local taxes and charges		356 (31.5%)	215 (45.8%)	151 (67.7%)	46 (86.8%)	158.13	3	.000*	.29
Local fees or related arrears divided into instalments	Real property tax	128 (11.3%)	87 (18.6%)	75 (33.6%)	25 (47.2%)	105.26	3	.000*	.24
	Tax on means of transport	56 (5%)	31 (6.6%)	26 (11.7%)	6 (11.3%)	16.49	3	.001*	.09
	Agricultural tax	58 (5.1%)	18 (3.8%)	2 (0.9%)	3 (5.7%)	8.64	3	.035*	.07
	Forest tax	21 (1.9%)	8 (1.7%)	2 (0.9%)	2 (3.8%)	2.28	3	.517	.04
	Marketplace fee	11 (1%)	3 (0.6%)	2 (0.9%)	2 (3.8%)	4.93	3	.177	.05
	Local charge	9 (0.8%)	2 (0.4%)	1 (0.4%)	2 (3.8%)	7.50	3	.057	.06
	Climate fee	8 (0.7%)	2 (0.4%)	0 (0%)	1 (1.9%)	3.34	3	.341	.04
	Advertising fee	7 (0.6%)	2 (0.4%)	0 (0%)	1 (1.9%)	3.29	3	.350	.04
	Dog owners’ fee	8 (0.7%)	3 (0.6%)	0 (0%)	1 (1.9%)	2.81	3	.421	.04

Throughout the text: *χ*^*2*^- Chi- square statistics, *df*- number of degrees of freedom, *p*- level of statistical significance (*p* < .05 is considered to be a statistically significant result; *p* < .05 – weak relationship, *p* < .01 – moderate relationship; *p* < .001 – strong relationship); V- Cramer’s V strength of association (V < .05 weak relationship .05 < V < .15 means moderate relationship; V > .15 means strong relationship).

*statistical results that meet the tests of significance

Source: Authors’ study

The authors analyzed the correlation between the administrative type of the municipality and the types of revenue instruments other than local taxes and charges. The analysis was conducted using Pearson *χ*^*2*^ tests. Table 9 revealed that municipalities differed in terms of releasing tenants of municipal premises from the obligation to pay rent for residential *p* < .01 and commercial premises *p* < .001. Such exemptions were most often used in cities with district rights. In these cities, lessees of municipal land were also significantly more often released from the obligation to pay rent (p < .001). A similar situation applied to subsidies on bills (p < .05), deferring the payment of rent, leases and fees for the use of municipal land (p < .001) and the release from payment for kindergartens and public nurseries (p < .001). Such preferences were least often used in rural municipalities.

**Table 9:** Correlations between the type of municipality and the type of non-tax revenue instruments used

Specification		Rural municipality	Urban and rural municipality	Urban municipality	Cities with district rights	*χ*^*2*^	*df*	*P*	*V*
Tenants of community premises released from the obligation to pay rent	Residential premises	19 (1.7%)	18 (3.8%)	7 (3.1%)	4 (7.5%)	12.13	3	.007*	.08
	Commercial premises	212 (18.8%)	150 (32.0%)	78 (35.0%)	28 (52.8%)	69.29	3	.000*	.19
Lessees of municipal land released from rent	Natural persons	50 (4.4%)	53 (11.3%)	45 (20.2%)	20 (37.7%)	119.72	3	.000*	.25
	Legal entities and organizational units without legal personality	40 (3.5%)	37 (7.9%)	34 (15.2%)	21 (39.6%)	130.48	3	.000*	.26
Limitation of the fees for using municipal infrastructure	Water supply fee	10 (0.9%)	7 (1.5%)	3 (1.3%)	2 (3.8%)	4.36	3	.225	.04
	Waste management fee	9 (0.8%)	4 (0.9%)	2 (0.9%)	2 (3.8%)	5.01	3	.171	.05
	Subsidies on bills	8 (0.7%)	4 (0.9%)	0 (0%)	2 (3.8%)	8.32	3	.040*	.07
Civil law liabilities due to the municipality deferred or divided into instalments	Rents	86 (7.6%)	61 (13%)	62 (27.8%)	22 (41.5%)	114.56	3	.000*	.25
	Leases	57 (5%)	53 (11.3%)	50 (22.4%)	20 (37.7%)	119.04	3	.000*	.25
	Fee for the use of municipal land	56 (5%)	43 (9.2%)	45 (20.2%)	16 (30.2%)	89.26	3	.000*	.22
Exemptions from the obligation to pay fixed fees for kindergartens and public nurseries for residents	Kindergartens	84 (7.4%)	70 (14.9%)	45 (20.2%)	15 (28.3%)	55.22	3	.000*	.17
	Public nurseries	69 (6.1%)	72 (15.4%)	54 (24.2%)	33 (62.3%)	197.96	3	.000*	.33

Source: Authors’ study

Then, the correlation between the size of the municipality (expressed as population), its current revenue per capita and the expenses on counteracting COVID-19 and the use of non-tax revenue instruments were analyzed. The Kendall tau correlation analysis (Table 10) demonstrated that all these factors influenced the use of such instruments as: releasing tenants from the payment of rent for premises, releasing lessees from the payment of rent for land, deferral of civil law liabilities due to the municipality and exempting residents from the payment of fixed fees for kindergartens and public nurseries. The correlations were positive, which means that these revenues were more often used in municipalities with a larger population, higher current revenue per capita, but also those that spent more funds on actions to counteract COVID-19. The correlation between the scope and diversity of the instruments used and the population was the strongest: municipalities with a higher number of residents used more diversified non-tax revenue instruments and they used such instruments more often.

**Table 10:** Correlations between the size of a municipality, its current revenues per capita and the expenditures on counteracting COVID-19 using non-tax revenue instruments (the Kendall tau correlation analysis)

Specification		Population	Current revenues per capita	Loss of revenue due to exemptions/allowances granted	Expenditures on actions related to COVID-19
Tenants of community premises released from the obligation to pay rent	Residential premises	.04*	−.01	.06**	.03
	Commercial premises	.21***	.06**	.14***	.16***
Lessees of municipal land released from rent	Natural persons	.19***	.08***	.19***	.16***
	Legal entities and organizational units without legal personality	.19***	.06**	.20***	.15***
Fees for using municipal infrastructure lowered	Water supply fee	.03	−.03	.05*	.03
	Waste management fee	.07**	.01	.01	.04
	Subsidies on bills	.04	.02	.01	.02
Civil law liabilities due to the municipality deferred or divided into instalments	Rents	.19***	.05*	.16***	.15***
	Leases	.19***	.07**	.17***	.16***
	Fee for the use of municipal land	.19***	.08***	.15***	.15***
Exemptions from the obligation to pay fixed fees for kindergartens and public nurseries for residents	Kindergartens	.17***	.07**	.11***	.14***
	Public nurseries	.28***	.11***	.19***	.23***

*p < .05; **p < .01; ***p < .001

Source: Authors’ study

**Fig. 3:**
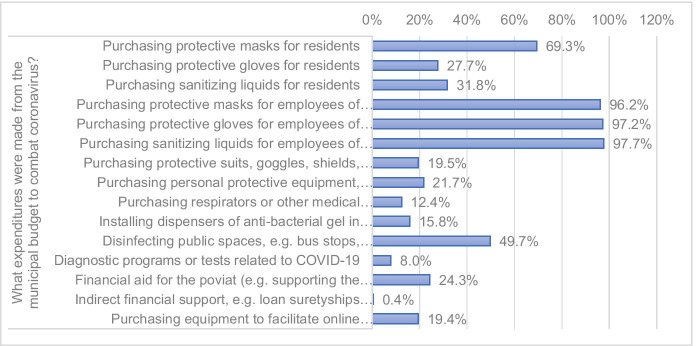
Expenses incurred from municipality budgets on combating COVID-19

The authors also analyzed issues related to expenditure instruments by identifying the directions of budget expenses incurred by municipalities for combating COVID-19. These expenses were usually related to the purchase of protective masks, gloves, and sanitizing liquids for employees of municipal offices and communal organizational units, followed by the costs of purchasing protective masks for residents and sanitizing public spaces (Fig. 3).

**Fig. 4:**
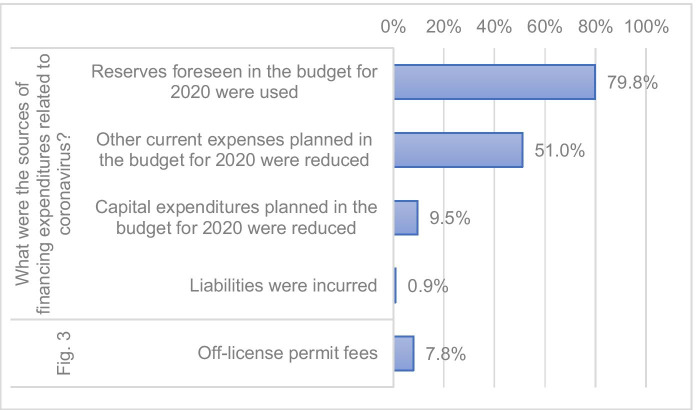
Sources of financing of expenses of municipalities on counteracting COVID-19

**Fig. 5:**
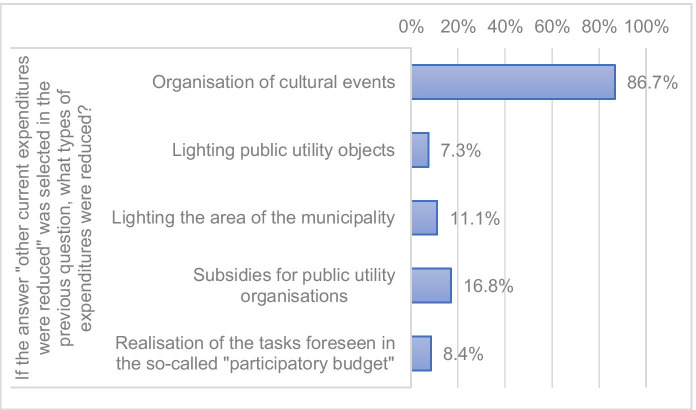
Reduction of current expenses of municipalities resulting from the need to finance activities to prevent COVID-19

Expenses related to counteracting COVID-19 were financed mainly from the reserves provided in the budget for 2020 (79.8%). More than half of municipalities (51.0%) reduced other expenses planned for 2020 in order to finance actions aimed at counteracting COVID-19. Such reductions usually applied to the organization of cultural events and to subsidies for public utility organizations. Municipalities also decided not to provide lighting of their area at night and suspended the financing of investment tasks planned by residents in the participatory budget formula (Fig. 4 and 5).

The expenditures of municipalities were aggregated into four categories, which were also linked to the type of municipality, in an attempt to find out whether the type had an influence on the diversification of expenditure instruments used to support business entities and residents in combating the pandemic (H2) and on the sources of financing these expenses (H3). The analysis was based on Pearson *χ*^*2*^ tests (Table 11). Considering the types of expenditures, it was only demonstrated that different municipalities used different policies for supporting hospitals *p* < .001. Cities with district rights incurred expenses on such support more often. On the other hand, no differences were found between different types of municipalities in terms of expenses on protective equipment (masks, gloves and sanitizing liquids) for residents, support for the district, and purchasing equipment to facilitate the organization of remote meetings of city council members and office employees. The municipalities did not differ in the aspect of sources of financing expenditures on counteracting COVID-19, either.

**Table 11:** Correlation between the type of municipality and the expenditures incurred on combating COVID-19 and the sources of their financing

Specification		Rural municipality	Urban and rural municipality	Urban municipality	cities with district rights	*χ*^*2*^	*df*	*p*	*V*
Type of expenses	Protective equipment for residents	789 (69.9%)	346 (73.8%)	171 (76.7%)	36 (67.9%)	5.91	3	.116	.06
	Support for hospitals	240 (21.3%)	161 (34.3%)	102 (45.7%)	31 (58.5%)	92.81	3	.000*	.22
	Financial aid for the district	275 (24.4%)	119 (25.4%)	55 (24.7%)	6 (11.3%)	5.17	3	.160	.05
	Equipment to facilitate the organization of remote meetings of city council members and office employees	227 (20.1%)	85 (18.1%)	42 (18.8%)	10 (18.9%)	0.90	3	.825	.02
Sources of financing the expenditures	Reserves foreseen in the budget for 2020	900 (79.7%)	373 (79.5%)	182 (81.6%)	38 (71.7%)	2.61	3	.456	.04
	Reducing other current expenses planned in the budget for 2020	582 (51.6%)	232 (49.5%)	108 (48.4%)	32 (60.4%)	3.03	3	.388	.04

Source: Authors’ study

## Discussion

The H1 hypothesis was partly confirmed. Tax exemptions, allowances and deferrals were most often used in urban municipalities and cities with district rights and the least often in rural municipalities. However, these differences were not very significant. Urban municipalities and cities with district rights usually divided real property tax and tax on means of transport into installments, while rural municipalities divided agricultural tax. These findings are not surprising and they may be explained by the specificity of the taxation base for these taxes because agricultural tax is charged mainly on arable land, which is usually located outside the borders of towns.

The administrative type of the municipality was a factor that differentiated the approach to releasing tenants from the obligation to pay rent. Such exemptions were most commonly used by cities with district rights. Thus, one may assume that the more rural the municipality, the less often it used non-tax revenue instruments. This may result from the specificity of the business activity conducted in rural municipalities, which have a lower number of service facilities and entities that provide hospitality or entertainment services. Other factors that differentiate the approach of municipalities to releasing tenants from rent are the size of the population, the amount of current revenue per capita, and even the expenses on counteracting the pandemic. These revenue instruments were more often used in cities with district rights, i.e. municipalities with a larger population and higher current revenue per capita, but also those that spent more funds on actions to counteract COVID-19. They also more often released their residents from the obligation to pay for kindergartens and public nurseries. The strongest correlation was found between the scope and variety of the instruments applied and the population. Such policy may be explained by the fact that cities with district rights are usually cultural and economic centers of their regions, so the businesses that suffered most in the pandemic are concentrated there. These municipalities have larger property resources that they may make available to private entities. These are also quite affluent municipalities, which are able to perform their statutory tasks in spite of the loss of revenues caused by such reductions.

- H2 assumed that the type of expenditure instruments used depends on the administrative type of the municipality. This assumption was confirmed only for support for hospitals. Expenses on such support were more often made by cities with district rights. No other statistically significant differences were found. However, it is worth noting that the number of rural municipalities which purchased electronic equipment for their authorities and their officials was rather high. The observed pattern may be explained as follows: the duties of cities with district rights include, among others, maintaining hospitals, which means that providing them with additional funds and purchasing new equipment belongs to the competencies of such municipalities. Other types of municipalities may also support hospitals, but it is a more time- and work-consuming process, as it requires taking the relevant resolutions.
- H3 assumed that the administrative type of the municipality influences the sources of financing the increased budget expenses related to counteracting the effects of COVID-19. This hypothesis was disproven. Municipalities did not differ in terms of the sources of financing such expenditures. This results from the fact that the pandemic crisis was unforeseen. However, the authors suppose that a similar research project conducted at the beginning of 2021 might reveal that cities with district rights and municipalities with higher revenues financed these expenses from loans and municipalities of all administrative types – from additional subsidies from the State Budget. On the other hand, some of the costs of the anti-crisis policy may be transferred to entities that provide services, e.g. sports clubs, or directly to residents.

## Conclusions

The COVID-19 crisis is a completely new phenomenon that gave rise to a new approach to counteracting crisis through budget revenues and expenditures. Already in the first months of the pandemic, municipalities took the effort to adapt local communities to function in the sanitary regimen and took some anti-crisis actions, although it is difficult to state that their activities formed a consistent anti-crisis strategy. First municipalities attempted to ensure sanitary protection for their residents by changing the conditions of work of self-government entities and their officials and attempted to create favorable conditions for remote education. Secondly, they supported small entrepreneurs and residents. However, one cannot say that these actions were financial strategies. They were taken *ad hoc* and most likely inspired by actions taken by other municipalities.

Few municipalities used tax exemptions. More often, real property tax was divided into installments on request of the taxpayers. Also, few municipalities lowered the fees for water consumption and waste disposal or subsidized utility bills. The payment of municipal receivables for the use of municipal land and presidents (residential and commercial ones) was deferred or divided into installments. Cities with district rights also abstained from enforcing the outstanding payments for the use of such premises.

Polish municipalities did not incur any expenses on the treatment of ill people or the activities of sanitary and epidemiological services, because the whole healthcare system is financed from state sources. Moreover, municipalities do not perform any tasks that would involve increased expenditures in pandemic conditions (apart from managing hospitals in cities with district rights), so they only incurred expenses on the promotion of health. Nearly all municipalities purchased protective masks and gloves as well as disinfection liquids for local government officials. Almost 70% of them also purchased masks for residents and half of the municipalities disinfected public spaces.

The scale of revenue loss and increase in expenditures was not large. Most of the municipalities declared a decrease in revenues due to granted exemptions, allowances, discounts and other activities aimed at counteracting the effects of COVID-19 on a level below 199 thousand USD. The extraordinary expenses connected with the pandemic were also usually below 199 thousand USD.

Expenses on counteracting COVID-19 were financed mainly from the reserves provided in the budget for 2020. The municipalities also reduced other, unnecessary current expenses: on cultural events and subsidies for public utility organizations. They also decided not to provide lighting of their area at night and stopped the financing of current and investment tasks proposed by residents in the participatory budget formula.

March and April 2020 were characterized by a high level of insecurity, both concerning the pandemic itself, its course, effects, as well as to decisions that would be made on the central level that might affect the activities of municipalities. A much more precise diagnosis of the influence of the COVID-19 pandemic on the functioning of local communities and a deeper reflection on the possibility to support recovery from the crisis is expected to emerge in the third quarter of 2020, after the end of the survey.
